# Gender-specific association between blood cell parameters and hyperuricemia in high-altitude areas

**DOI:** 10.3389/fpubh.2024.1336674

**Published:** 2024-03-25

**Authors:** Danli Cui, Ruoying Huang, Dexi Yongzong, Bo Lin, Xia Huang, Qimei Ciren, Xuelian Zhou

**Affiliations:** ^1^The People’s Hospital of Dazu, Chongqing, China; ^2^The People’s Hospital of Chaya County, Changdu, Tibet, China; ^3^Chongqing Blood Center, Chongqing, China

**Keywords:** hyperuricemia, high-altitude areas, gender differences, metabolic dynamics, blood cell parameters

## Abstract

**Background:**

Hyperuricemia is a common metabolic disorder linked to various health conditions. Its prevalence varies among populations and genders, and high-altitude environments may contribute to its development. Understanding the connection between blood cell parameters and hyperuricemia in high-altitude areas can shed light on the underlying mechanisms. This study aimed to investigate the relationship between blood cell parameters and hyperuricemia in high-altitude areas, with a particular focus on gender differences.

**Methods:**

We consecutively enrolled all eligible Tibetan participants aged 18–60 who were undergoing routine medical examinations at the People’s Hospital of Chaya County between January and December 2022. During this period, demographic and laboratory data were collected to investigate the risk factors associated with hyperuricemia.

**Results:**

Among the participants, 46.09% were diagnosed with hyperuricemia. In the male cohort, significant correlations were found between serum uric acid (SUA) levels and red blood cell (RBC) count, creatinine (Cr). Urea, alanine transaminase (ALT), and albumin (ALB). Notably, RBC exhibited the strongest association. Conversely, in the female cohort, elevated SUA levels were associated with factors such as white blood cell (WBC) count. Urea, ALT, and ALB, with WBC demonstrating the most significant association. Further analysis within the female group revealed a compelling relationship between SUA levels and specific white blood cell subtypes, particularly neutrophils (Neu).

**Conclusion:**

This study revealed gender-specific associations between SUA levels and blood cell parameters in high-altitude areas. In males, RBC count may play a role in hyperuricemia, while in females, WBC count appears to be a significant factor. These findings contribute to our understanding of metabolic dynamics in high-altitude regions but require further research for comprehensive mechanistic insights.

## 1 Introduction

Hyperuricemia, characterized by elevated levels of serum uric acid (SUA), is a common metabolic disorder worldwide (1). This condition occurs when there is an imbalance between the production and excretion of uric acid, leading to its accumulation in the body. Hyperuricemia is clinically significant as it is associated with the development of various health conditions, including gout, type 2 diabetes, chronic kidney disease, hypertension, cardiovascular diseases, and thyroid dysfunction (2–5). These associations highlight the importance of understanding and managing hyperuricemia in order to prevent and treat these related health conditions.

The level of SUA is influenced by various factors such as age, gender, race, genetics, dietary habits, medication, and environment (6–8). In recent years, there has been an increasing trend in the prevalence of hyperuricemia, with higher rates among males compared to females (7). Previously considered a condition primarily affecting wealthy white men, recent evidence has revealed that gout is more prevalent among racial/ethnic minorities, indigenous populations, and other marginalized groups (9). This has led to significant health disparities in these communities. Additionally, environmental factors also play a role in the development of hyperuricemia (10).

High-altitude areas are known for their unique environmental conditions, including low-pressure, low-temperature, and hypoxic conditions (11). These conditions can lead to an increase in oxidative cellular damage and trigger various metabolic and physiological changes (12). In response to these stimuli, the human body undergoes specific changes (13), which can be observed through variations in blood cell parameters (14). For instance, one of the variations is the increased production of red blood cells (RBC) in order to compensate for low oxygen levels and ensure adequate oxygen supply to the tissues (15). These adaptations are vital for survival in high-altitude environments. However, it is crucial to investigate whether these variations in blood cell parameters are associated with the development of hyperuricemia in individuals residing in high-altitude areas. Understanding the potential link between blood cell parameters and hyperuricemia in high-altitude regions can provide valuable insights into the underlying mechanisms of this metabolic disorder.

Therefore, the main objective of this study was to investigate the association between blood cell parameters and hyperuricemia in high-altitude areas through a population-based study. Specifically, we aimed to explore if there were gender differences in the relationship between blood cell parameters and hyperuricemia. By conducting this study, we aimed to contribute to the understanding of the factors influencing hyperuricemia in high-altitude areas, which could have implications for the prevention and management of this condition in these regions.

## 2 Materials and methods

### 2.1 Research cohort description

**Figure 1:**
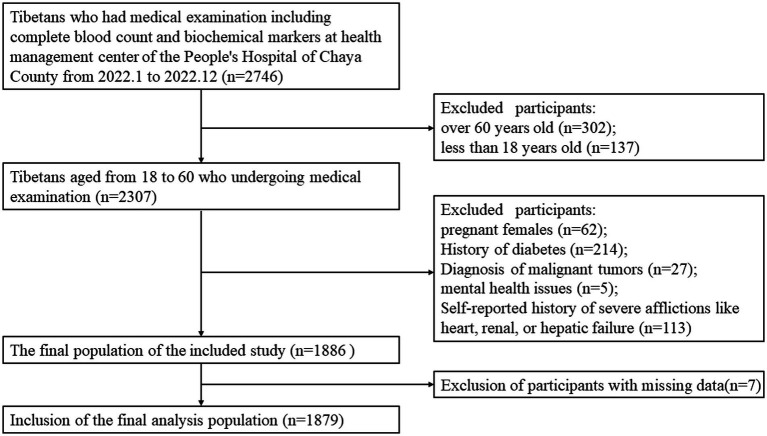
The flow chart of the study.

We conducted a retrospective cross-sectional research study involving the Tibetan population in Chaya County (at an average altitude of approximately 3,500 m), within the age group of 18–60 years, who were part of the regular health check-up at the People’s Hospital of Chaya County. This institution is a Grade 2B hospital situated at an average altitude of roughly 3,500 m, which serves a population of approximately 80,000 people, with over 80% of them being local residents. Our study population was gathered over the course of the year 2022. The exclusion criteria incorporated pregnant females, individuals with a diagnosis of malignant tumors, mental health issues, or severe afflictions like heart, renal, or hepatic failure, as indicated either by medical documentation or self-reported diagnosis (Figure 1). All information related to participants was anonymized. Due to the retrospective nature of this study, obtaining informed consent from patients is not required when analyzing the data.

### 2.2 Data collection and laboratory analysis procedures

Data were collected in strict accordance with the medical examination protocols of the People’s Hospital of Chaya County. Trained healthcare professionals obtained venous blood samples (2 mL for complete blood count and an additional 3–4 mL for biochemical assessment) from participants who had fasted overnight. These samples were then transferred to the laboratory at the People’s Hospital of Chaya County for further examination.

The parameters for the complete blood count were determined using an XN-550 automated hematology analyzer, a device by SYSMEX, Kobe, Japan. Additionally, biochemical markers, encompassing SUA, Urea, creatinine (Cr), alanine aminotransferase (ALT), globulin (GLB), albumin (ALB), were analyzed using a BS-800 M auto-analyzer, a machine developed by Mindray, China.

### 2.3 Diagnostic criteria and grouping description

In this study, the diagnosis of hyperuricemia was made based on pre-established criteria, which entailed SUA concentrations exceeding 420 μmol/L in men or 360 μmol/L in females. Participants were then divided into two groups: the hyperuricemia group (HUA) and the normouricemia group (NUA), according to these aforementioned criteria.

### 2.4 Statistical analysis

Statistical analysis was conducted using SPSS 23.0 software. The chi-square test was employed to analyze categorical data, such as gender, in order to assess any significant differences. T-test were used to analyze count data. A multivariable binary logistic regression (adjusted odds ratios [OR^a^], confidence interval [CI]) was performed to assess the relationship between hyperuricemia prevalence and blood parameters. Furthermore, multivariate linear regression analysis was performed to explore the relationship between white blood cell (WBC) count and SUA levels in females. Data visualization and graphical representations were created using Graphpad Prism 9.0 software.

## 3 Results

### 3.1 Baseline characteristics of the participants

A total of 1879 consecutive general adults (986 males and 893 females) with an average age of 35.27 ± 9.92 years (ranging from 18 to 60 years old), were included from Tibetans undergoing medical examination at the Health Management Center. The baseline characteristics of the participants in the NUA and HUA groups, segregated by gender, are presented in Table 1. Among the participants, 866 (46.09%) individuals were diagnosed with hyperuricemia based on the diagnostic criteria. There were significant differences in the levels of SUA, Urea, Cr, ALB, RBC count, hemoglobin (Hb), and platelet count (PLT) (*p* < 0.05) for all cases. In males, significant differences were observed in ALT and GLB levels, while in females, there was a significant difference in WBC count between the NUA and HUA group.

**Table 1:** Baseline characteristics of participants in the NUA and HUA groups by gender.

Parameters	Total	Male			Female		
		NUA	HUA	*p*	NUA	HUA	*p*
	(*n* = 1879)	(*n* = 533)	(*n* = 453)		(*n* = 480)	(*n* = 413)	
Age (year)	35.27 ± 9.92	37.82 ± 11.06	36.7 ± 9.56	0.089	31.93 ± 5.65	32.99 ± 10.81	0.073
SUA (μmol/L)	381.26 ± 95.26	346.36 ± 57.25	500.23 ± 60.73	<0.001	285.58 ± 44.53	406.99 ± 48.09	<0.001
Urea (mmol/L)	4.94 ± 2.13	5.01 ± 1.82	5.59 ± 2.62	<0.001	4.38 ± 1.26	4.8 ± 2.49	0.002
Cr (μmol/L)	74.94 ± 18.45	76.68 ± 11.48	84.14 ± 21.58	<0.001	66.89 ± 11.94	71.96 ± 22.87	<0.001
ALT (U/L)	35.99 ± 31.92	40.91 ± 34.01	54.6 ± 36.84	<0.001	22.42 ± 17.76	25 ± 23.39	0.067
GLB (g/L)	28.51 ± 4.23	28.38 ± 4.59	27.84 ± 3.9	0.045	29.01 ± 3.52	28.82 ± 4.75	0.515
ALB (g/L)	46.14 ± 5.67	46.01 ± 5.34	48.47 ± 4.14	<0.001	47.98 ± 3.35	41.6 ± 6.89	<0.001
WBC (×10^9^/L)	7.23 ± 3.18	7.42 ± 3.27	7.08 ± 2.59	0.073	5.9 ± 1.61	8.7 ± 4.19	<0.001
Neu (×10^9^/L)	4.72 ± 3.11	4.87 ± 3.15	4.35 ± 2.47	0.004	3.46 ± 1.37	6.38 ± 4.19	<0.001
Lym (×10^9^/L)	1.9 ± 0.67	1.86 ± 0.76	2.05 ± 0.69	<0.001	1.95 ± 0.5	1.75 ± 0.67	<0.001
Mon (×10^9^/L)	0.45 ± 0.2	0.51 ± 0.23	0.49 ± 0.2	0.312	0.36 ± 0.11	0.45 ± 0.19	<0.001
Eos (×10^9^/L)	0.12 ± 0.34	0.15 ± 0.61	0.14 ± 0.12	0.661	0.1 ± 0.08	0.09 ± 0.1	0.109
Bas (×10^9^/L)	0.04 ± 0.02	0.04 ± 0.02	0.04 ± 0.02	0.001	0.04 ± 0.02	0.03 ± 0.02	<0.001
RBC (×10^12^/L)	5.09 ± 0.76	5.39 ± 0.63	5.63 ± 0.69	<0.001	4.8 ± 0.37	4.44 ± 0.72	<0.001
Hb (g/L)	149.88 ± 26.28	161.26 ± 19.67	170.04 ± 19.88	<0.001	138.33 ± 16.12	126.5 ± 24.39	<0.001
PLT (×10^9^/L)	247.28 ± 75.53	246.82 ± 77.2	233.8 ± 72.57	0.007	267.36 ± 67.91	239.32 ± 80.2	<0.001

### 3.2 Binary logistic regression analysis shows factors associated with high SUA levels

**Figure 2:**
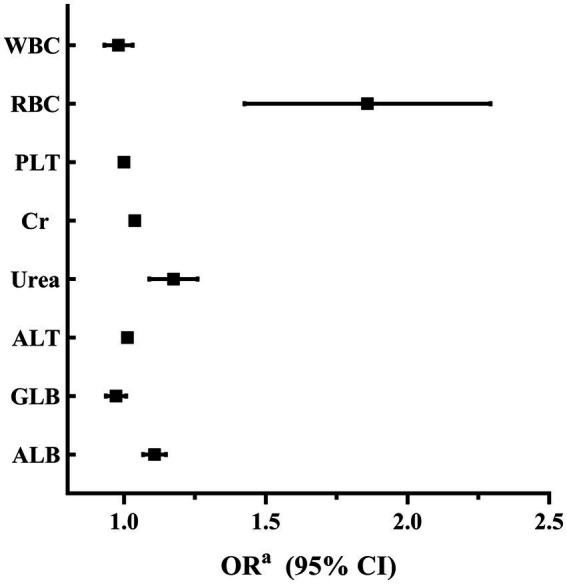
Factors associated with elevated levels of SUA in male participants.

**Figure 3:**
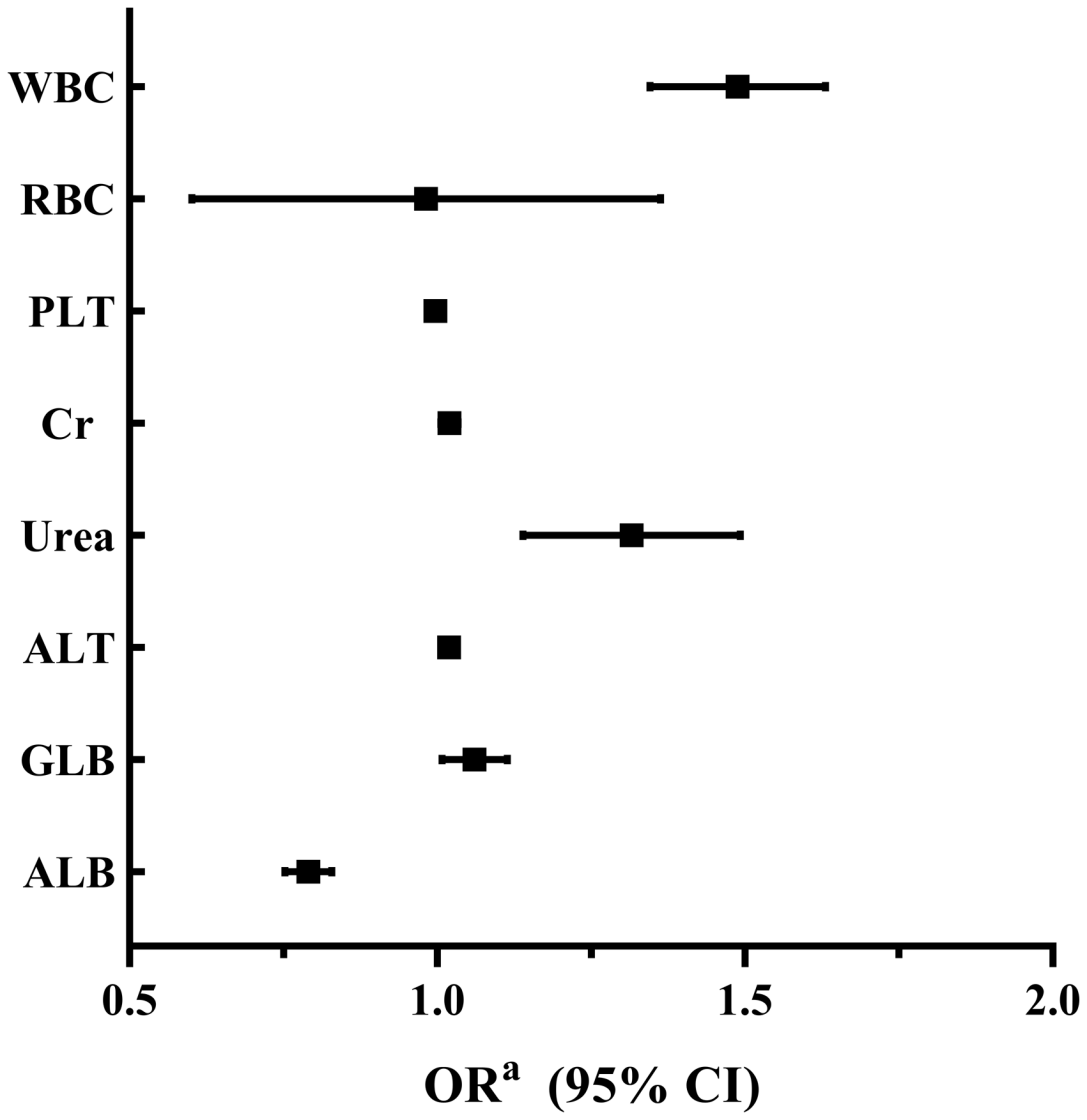
Factors associated with elevated levels of SUA in female participants.

A binary logistic regression analysis was performed to investigate the factors associated with high SUA levels in male and female participants. In male participants, the analysis revealed that levels of RBC, Cr, Urea, ALT, and ALB were independently associated with high SUA levels. Among these factors, RBC showed the strongest association with high SUA levels (adjusted OR 1.825, 95% CI 1.442–2.309; *p* < 0.001). These findings are summarized in Table 2 and Figure 2. In female participants, the analysis indicated that WBC, Urea, ALT, and ALB were independently associated with high SUA levels. WBC had the highest association with high SUA levels among these factors (adjusted OR 1.483, 95% CI 1.348–1.633; *p* < 0.001). The results for female participants are presented in Table 3 and Figure 3.

**Table 2:** Factors associated with high SUA levels in male participants.

	Male				
	*B*	Wald χ^2^	*p* value	OR^a^	95% CI
WBC	−0.022	0.689	0.406	0.979	0.930–1.030
RBC	0.601	25.062	<0.001	1.825	1.442–2.309
PLT	−0.001	0.636	0.425	0.999	0.997–1.001
Cr	0.036	38.408	<0.001	1.037	1.025–1.049
Urea	0.158	18.156	<0.001	1.172	1.089–1.260
ALT	0.011	24.004	<0.001	1.011	1.007–1.015
GLB	−0.030	2.528	0.0012	0.970	0.935–1.007
ALB	0.101	29.943	<0.001	1.107	1.067–1.147

^a^Adjusted for age.

**Table 3:** Factors associated with high SUA levels in female participants.

	Female				
	*B*	Wald χ^2^	*p* value	OR^a^	95% CI
WBC	0.394	64.918	<0.001	1.483	1.348–1.633
RBC	0.069	0.118	0.731	0.933	0.628–1.385
PLT	−0.003	5.710	0.017	0.997	0.994–0.999
Cr	0.019	7.858	0.005	1.020	1.006–1.034
Urea	0.268	15.316	<0.001	1.308	1.143–1.496
ALT	0.019	14.912	<0.001	1.019	1.009–1.029
GLB	0.058	5.259	0.022	1.060	1.008–1.114
ALB	−0.236	93.941	<0.001	0.790	0.753–0.829

^a^Adjusted for age.

### 3.3 Association between WBC subtypes and SUA levels in female participants

In the analysis of female participants, a multivariate linear regression was performed to investigate the relationship between WBC subtypes, including Neutrophils (Neu), Lymphocytes (Lym), Monocytes (Mon), Eosinophils (Eos), and Basophils (Bas), and SUA levels. The results revealed a strong association between Neutrophils (Neu) and high SUA levels in Table 4.

**Table 4:** Association between WBC subtypes and SUA levels in female participants.

	*B*	*β*	*t*	*p* value	*F*
Neu	6.540	0.288	6.762	<0.001	26.586^***^
Lym	−2.122	−0.017	−0.476	0.634	
Mon	36.233	0.076	1.820	0.069	
Eos	60.937	0.071	1.961	0.050	
Bas	−488.117	−0.127	−3.514	<0.001	

***Means *p*<0.001.

## 4 Discussion

Our study aimed to investigate the association between SUA levels, blood cell counts, and gender in individuals residing in high-altitude areas. Our study has revealed a significant correlation between SUA levels and various blood parameters. Notably, we have observed gender-specific disparities in these connections. In the male cohort, the relationship between SUA levels and RBC was found to be the most pronounced. Conversely, in the female cohort, the greatest association was found between SUA levels and WBC.

Previous studies have shown regional and population disparities in the incidence of hyperuricemia in China (16, 17). Notably, the Tibetan population in high-altitude areas exhibits different rates compared to the Han population in lowland regions (18, 19). A multicenter study involving a large sample size demonstrated that Tibetan males have a higher incidence of hyperuricemia compared to Han males, while Tibetan females have a lower incidence of hyperuricemia than Han females (18). This gender-related divergence in uric acid alterations induced by high altitude warrants attention. However, the precise causes and mechanisms underlying these trends remain elusive.

Within the male cohort, our findings reveal a significant correlation between SUA levels and various factors, including RBC, Cr, Urea, ALT, and ALB. Among these factors, RBC demonstrates the strongest association. It is well-established that individuals residing in high-altitude regions exhibit an increased production of red blood cells as an adaptive response to the limited oxygen environment (15). As RBCs have a limited lifespan (20), their breakdown releases purine nucleotides, uric acid precursors (21). Accumulation of these byproducts, when converted into uric acid in the liver, leads to elevated SUA levels (21, 22). Since RBC count is higher in males, more RBCs are destroyed over the same time period, resulting in a higher release of uric acid precursors. Therefore, this variation may provide an explanation for the stronger correlation between male hyperuricemia and RBC count. Conversely, within the female cohort, we observed associations between elevated SUA levels and factors such as WBC, Urea, ALT, and ALB, with WBC demonstrating the most significant association. Further exploration within the female group revealed a compelling relationship between SUA levels and specific white blood cell subtypes, particularly Neu. WBC is an indicator of immune responses and inflammation (23). In high-altitude regions, the hypoxic conditions tend to suppress the immune response, leading to a decrease in WBC counts, including Neu (24). Therefore, the lower prevalence of hyperuricemia in females at high altitudes may be attributed to the suppressive effects of hypoxic conditions on WBC counts.

The observed gender differences in the association between blood cell counts and SUA levels on the plateau raise interesting questions about the underlying mechanisms. It is possible that hormonal differences between males and females play a role in modulating these associations. For example, testosterone levels may influence RBC production (25), consequently, affect SUA levels in males. On the other hand, estrogen levels may influence WBC production (26), therefore, impact SUA levels in females. Another potential explanation for the observed gender differences could be genetic factors. There may be genetic variations that affect the response of RBC and WBC counts to the hypoxic environment on the plateau, leading to different effects on SUA levels in males and females. Investigating the impact of hormones and genetic factors on blood cell counts and SUA levels in both genders would provide valuable insights into these potential mechanisms.

There are a few limitations to note. Firstly, the study solely focused on blood test results without considering crucial factors such as BMI, lifestyle, socio-economic status, and dietary intake. It is important to note that protein intake, in particular, plays a significant role in the development of Hyperuricemia. By not taking these factors into account, the study may overlook important contributors to the observed outcomes. Secondly, it only looked at the Tibetan population in high-altitude areas, without comparing them to lowland individuals, potentially limiting our understanding of altitude effects. Last but not the least, given that over 80% of the population being studied comprises local residents who have resided in the high-altitude region for a significant period, experiencing chronic hypoxia, it is important to consider the impact of this chronic hypoxia on blood cell parameters irrespective of gender. As a result, it may not be directly applicable to regions devoid of chronic hypoxia, such as low-altitude areas. Therefore, it is essential to exercise caution when attempting to generalize our findings to populations residing in non-hypoxic regions.

In conclusion, our study provides valuable insights into the gender differences associated with SUA levels and blood cell counts in high-altitude areas. The results suggest that both red blood cells and white blood cells, may contribute to the gender-specific occurrence of hyperuricemia in high-altitude regions. However, it is important to acknowledge that this study offers only a preliminary exploration of these relationships, and further research is warranted to comprehensively understand the underlying mechanisms and identify potential treatment strategies for managing hyperuricemia in these populations.

## Data availability statement

The original contributions of the study are included in the article. For further inquiries, please contact the corresponding author.

## Ethics statement

Written informed consent was not obtained from the individual(s) for the publication of any potentially identifiable images or data included in this article because due to the retrospective nature of this study, obtaining informed consent from patients is not required when analyzing the data.

## Author contributions

DC: Conceptualization, Data curation, Formal analysis, Methodology, Software, Writing – original draft, Writing – review & editing. RH: Formal analysis, Investigation, Software, Writing – original draft. DY: Data curation, Investigation, Resources, Software, Writing – original draft. BL: Data curation, Investigation, Methodology, Resources, Writing – original draft. XH: Conceptualization, Project administration, Supervision, Validation, Visualization, Writing – review & editing. QC: Conceptualization, Project administration, Supervision, Validation, Visualization, Writing – review & editing. XZ: Conceptualization, Funding acquisition, Project administration, Resources, Supervision, Validation, Visualization, Writing – review & editing.
